# Mechanisms Protecting *Acinetobacter baumannii* against Multiple Stresses Triggered by the Host Immune Response, Antibiotics and Outside-Host Environment

**DOI:** 10.3390/ijms21155498

**Published:** 2020-07-31

**Authors:** Soroosh Monem, Beata Furmanek-Blaszk, Adrianna Łupkowska, Dorota Kuczyńska-Wiśnik, Karolina Stojowska-Swędrzyńska, Ewa Laskowska

**Affiliations:** 1 Department of General and Medical Biochemistry, Faculty of Biology, University of Gdansk, Wita Stwosza 59, 80-308 Gdansk, Poland; soroosh.monem@phdstud.ug.edu.pl (S.M.); adrianna.lupkowska@phdstud.ug.edu.pl (A.Ł.); dorota.kuczynska-wisnik@ug.edu.pl (D.K.-W.); karolina.stojowska-swedrzynska@ug.edu.pl (K.S.-S.); 2 Department of Microbiology, Faculty of Biology, University of Gdansk, Wita Stwosza 59, 80-308 Gdansk, Poland; beata.furmanek-blaszk@ug.edu.pl

**Keywords:** *Acinetobacter baumannii*, biofilm, desiccation stress, multidrug resistance, persisters, proteostasis

## Abstract

*Acinetobacter baumannii* is considered one of the most persistent pathogens responsible for nosocomial infections. Due to the emergence of multidrug resistant strains, as well as high morbidity and mortality caused by this pathogen, *A. baumannii* was placed on the World Health Organization (WHO) drug-resistant bacteria and antimicrobial resistance research priority list. This review summarizes current studies on mechanisms that protect *A. baumannii* against multiple stresses caused by the host immune response, outside host environment, and antibiotic treatment. We particularly focus on the ability of *A. baumannii* to survive long-term desiccation on abiotic surfaces and the population heterogeneity in *A. baumannii* biofilms. Insight into these protective mechanisms may provide clues for the development of new strategies to fight multidrug resistant strains of *A. baumannii*.

## 1. Introduction

Gram-negative coccobacillus *Acinetobacter baumannii* belongs to a group of ESKAPE pathogens. ESKAPE is the acronym for the group of bacteria that include *Enterococcus faecium*, *Staphylococcus aureus*, *Klebsiella pneumoniae*, *A. baumannii*, *Pseudomonas aeruginosa*, and *Enterobacter* spp. Due to their ability to effectively *escape* antibiotic treatments, these multidrug-resistant (MDR) pathogens are common causes of life-threatening infections affecting mainly immunocompromised and critically ill patients in intensive care units (ICUs) [1]. In recent years, the overall number of antibiotics that act on the ESKAPE pathogens decreased significantly [1,2]. In 2017, the World Health Organization (WHO) published a list of 12 “priority” pathogens encompassing the ESKAPE group, for which new antibiotics are urgently needed. The WHO classifies carbapenem-resistant *A. baumannii* as the number one critical pathogen. Major risk factors for the acquisition of *A. baumannii* include antibiotic usage, especially β-lactams—the most commonly used drugs to treat infections caused by important pathogens which cause a variety of diseases in humans and animals. The second most common risk factor is mechanical ventilation, while other risks include surgical wound infections and invasive procedures such as central venous or urinary catheters [3]. It was demonstrated that approximately 1000,000 people globally are infected with *A. baumannii* every year, while the emergence of MDR strains is reported worldwide [4,5,6]. Antimicrobial treatment of MDR *A. baumannii* infections include colistin, sulbactam, and tigecycline, used in combination with other antibiotics [1,7,8]. A recently published global study, the Tigecycline Evaluation and Surveillance Trial (TEST), revealed that the percentage of MDR *A. baumannii* isolates was the highest among all analyzed Gram-negative bacteria, and it increased from 23% in 2004 to 63% in 2014 [4]. *A. baumannii* causes a range of infections, including ventilator-associated pneumonia, bacteremia, meningitis, urinary tract, wound, and bone infections [2,9]. The risk of mortality is high and often reaches 40–50% in ICU [10,11]. *A. baumannii* is a life-threatening problem not only because of multidrug resistance but also its ability to evade the host immune response and survive under harsh environmental conditions. In this review, we present various mechanisms that protect *A. baumannii* against the innate host immune response and stresses caused by the outside host environment. We focus on (1) the ability of *A. baumannii* to survive long-term desiccation, (2) factors involved in maintaining proteome homeostasis in *A. baumannii* cells, (3) the population heterogeneity in *A. baumannii* biofilms, and (4) the mechanism underlying *A. baumannii* antibiotic resistance.

## 2. *A. baumannii* and the Host Innate Immune Response

### 2.1. The First Line of Host Defense against A. baumannii

Neutrophils, macrophages, antimicrobial peptides (AMPs), and complement system components are the first line of innate immune defense that *A. baumannii* encounters during infection. Neutrophils can kill bacteria via phagocytosis, degranulation, or NETosis—a specific type of cell death pathway resulting in the release of the neutrophil extracellular traps (NETs). In NETs, chromatin forms a web-like structure decorated with antibacterial factors, including neutrophil elastase and AMPs [9]. Multiple studies suggest that neutrophils play a crucial role in the control of *A. baumannii* infection [12,13,14,15]; however, contradictory results showing that neutrophils do not kill *A. baumannii* were also reported [16,17]. After phagocytosis, neutrophil clearance of *A. baumannii* is mainly dependent on reduced nicotinamide adenine dinucleotide phosphate (NADPH) oxidase which generates reactive oxygen species (ROS) to kill the pathogen [12]. The stimulation of H_2_O_2_ production in the lung of a mouse model in response to *A. baumannii* infection confirmed this observation [18]. It was also found that *A. baumannii* inhibits NETosis, in contrast to other Gram-negative bacteria that trigger NET formation [17,19,20]. The role of macrophages in eliminating *A. baumannii* remains controversial. Most studies showed that macrophages play a minor role during *A. baumannii* infection [14,15,21]. However, Qiu et al. demonstrated that macrophages could be the first line of defense against respiratory *A. baumannii* infections; the depletion of alveolar macrophages significantly enhanced the susceptibility of mice to *A. baumannii* [22]. In addition, phagocytosis and killing of *A. baumannii* were observed in vitro, and the macrophages produced nitric oxide and secreted proinflammatory cytokines and chemokines [22].

AMPs are expressed constitutively or induced in different types of cells and tissues. Most of the AMPs are cationic; therefore, they can easily target the negatively charged surface of bacteria [23,24]. To date, 139 human host defense peptides were identified [25]. Cathelicidin-derived LL-37 is the best-studied AMP that kills *A. baumannii* cells through binding to the outer membrane protein A (OmpA) [24,26]. The LL-37 precursor, human cationic antibacterial peptide (hCAP-18), is produced by epithelial cells and neutrophils [13]. Recently, it was demonstrated that LL-37 forms a dimer of two anti-parallel amphipathic α-helices without supercoiling [27]. LL-37 helices target and extract lipopolysaccharides (LPS) to form holes in the outer membrane (OM). After diffusion into the periplasmic space, LL-37 may extract lipids from the inner membrane, forming a fibril-like structure [27]. Two other AMPs that belong to the human beta defensins, hBD-2 and hBD-3, were shown to kill *A. baumannii* in a concentration-dependent manner [28].

Another element of the immune system directed against *A. baumannii* involves the complement system components. The complement system consists of more than 30 plasma proteins that collaborate as a cascade triggering either bacterial cell lysis or opsonization and phagocytosis [29]. There are three pathways of complement activation: classical, lectin, and alternative pathways. The classical pathway is initiated by immune complexes, whereas the lectin pathway is triggered by pathogen-specific carbohydrates. The alternative pathway, which is responsible for the killing of *A. baumannii* [30,31], is permanently active due to spontaneous hydrolysis of the central complement system component, C3, enabling fast detection of pathogens [29,32]. Several studies reported that the depletion of the complement results in an increase of *A. baumannii* viability in human serum or a mouse model of *A. baumannii* infection [15,33,34].

### 2.2. Mechanisms Protecting A. baumannii against the Innate Immune Response

*A. baumannii* uses different virulence factors or mechanisms to evade the innate immune response. Surface glycoconjugates play key roles, but other strategies, including secreted proteins and metabolic pathways, also participate in the defense against the immune system [35,36].

The first barrier that protects *A. baumannii* against the immune host response is an exopolysaccharide capsule. The capsule is formed from long-chain polysaccharides composed of repeated carbohydrate units (K units). The synthesis of capsular polysaccharides (CPS) is dependent on a K locus (KL), which contains genes for synthesis of activated sugar precursors, glycosyl transfer, glycan modification, and oligosaccharide repeat-unit processing [37]. To date, 128 KL gene clusters were identified in *A. baumannii* [38]. Therefore, the monosaccharide composition and CPS structure are highly variable in *A. baumannii* strains [37,39,40,41,42,43]. K units vary in length and may consist of two to six residues. Common neutral sugars such as d-glucose, d-galactose, *N*-acetyl-d-glucosamine, and *N*-acetyl-d-galactosamine or rare sugars, including derivatives of pseudaminic, legionaminic, or acinetaminic acid, can be incorporated into CPS. Interestingly, acinetaminic acid was never found in nature before its detection in *A. baumanni* isolates with K12 and K13 gene clusters [42]. The CPS of different isolates are linear or branched and may possess non-carbohydrate substituents, including the most frequent *O*- and *N*-acyl groups [44]. The Wzy pathway encoded by the K locus is responsible for the export and extracellular assembly of CPS [45]. The K locus is regulated by the two-component signal transduction system BfmRS. The global BfmR regulator, along with histidine kinase BfmS, controls a variety of processes, including biogenesis of *A. baumannii* envelope elements, formation of biofilms, desiccation tolerance, and multiple stress responses [46,47,48,49]. Several studies demonstrated that the production of capsules affects *A. baumannii* virulence and persistence in the host [50,51]. Capsule-enriched strains cause more severe disease or higher lethality [35,45,50,52,53]. These observations may be partly explained by the high hydrophilicity of CPS and negative charges of CPS monosaccharides that prevent phagocytosis by hindering interactions with the negatively charged surfaces of neutrophils and macrophages, preventing phagocytosis [35]. However, it should be noted that the highly variable structure of the CPS may affect its capacity as a protective barrier against the immune response and other stresses, including complement-mediated killing, lysozyme degradation, and ROS [45,50,51,52,53,54].

Another barrier protecting *A. baumannii* against the host response is the OM. LPS, the main component of the outer layer of the OM, contains three domains: (1) lipid A, the membrane anchor glycosylated with (2) a core oligosaccharide which may provide an attachment site for (3) a long-chain *O*-antigenic polysaccharide [35]. In *A. baumannii*, the primary component of the outer layer of the OM is lipooligosaccharide (LOS) which, in contrast to the typical LPS, lacks the *O*-antigen [37]. *A. baumannii* LOS belongs to the group of pathogen-associated molecular patterns (PAMPs) recognized by Toll-like receptor 4 (TLR4) [13,55]. TLR4 is one of the pattern recognition receptors (PRRs) which function as cell-surface sensors of bacterial infection. *A. baumannii* LOS triggers a TLR4-mediated release of tumor necrosis factor (TNF) and interleukin 8 (IL-8) from macrophages [56]. This inflammation response is beneficial for the host; however, if it is upregulated, it can result in a cytokine storm and septic shock [57]. In the case of highly virulent *A. baumannii*, enhanced TLR4 activation is correlated to increased shedding of LOS into growth medium [58]. *A. baumannii* constitutively synthesizes hepta-acylated lipid A, under standard growth conditions, in contrast to other Gram-negative bacteria that upregulate its synthesis only under stress conditions [59]. Constitutive hepta-acylation of lipid A fortifies the OM to protect *A. baumannii* from cationic AMPs, lysozyme, and colistin, which is the last-resort antibiotic to treat MDR *A. baumannii* infections [59]. Colistin resistance may result from a complete lack of LOS or the addition of galactosamine to LOS. LOS deficiency significantly alters the interaction of *A. baumannii* with the host innate immune system. The overall pro-inflammatory response to LOS-deficient *A. baumannii* is reduced due to the lack of TLR-4 mediated stimulation. Instead of the TLR4-dependent mechanism, the TLR2-dependent mechanism is activated [60]. This alternative response is probably a consequence of increased expression of specific lipoproteins, transporters, and other surface components that compensate for the lack of LOS [61]. Although LOS-deficient strains are colistin-resistant, they exhibit decreased virulence and increased susceptibility to LL-37 and lysozyme [60,62,63,64].

Apart from the surface glycoconjugates, essential virulence factors of *A. baumannii* are OM proteins. The best-characterized *A. baumannii* OM protein, OmpA porin, is responsible for adhesion and invasion of *A. baumannii* into human epithelial cells [65,66,67,68]. It was found that the overproduction of OmpA is a risk factor for the development of *A. baumannii* pneumonia and bacteraemia, as well as for an increased mortality rate [69]. Consistently, the deletion of the *ompA* gene reduced *A. baumannii* bacterial dissemination and development of secondary pneumonia in the murine peritoneal sepsis model [69]. OmpA is secreted and enters epithelial cells via outer membrane vesicles (OMVs) [70,71,72]. OMVs are used by Gram-negative bacteria to deliver toxins, virulence factors, and other effector molecules to host cells. After entering the epithelial cell, OmpA migrates to mitochondria and stimulates the release of cytochrome *c*, finally promoting apoptosis [65]. In addition, OmpA triggers cell death by inducing the expression of TLR2 and production of nitrogen oxide. Lee et al. found that high concentrations of OmpA induces ROS production, leading to early-onset apoptosis and delayed-onset necrosis in dendritic cells (DCs) [73]. These results demonstrate that OmpA can cause the death of DSc, thereby impairing T-cell responses against *A. baumannii*. Other studies revealed that the interaction of OmpA with the fluid-phase complement regulator factor H (FH) enables *A. baumannii* to escape complement and to survive in human serum [31]. Two other *A. baumannii* proteins, CipA and the protein killing factor (PKF) serine protease, also contribute to serum resistance. The recently identified CipA is a plasminogen-binding protein exposed on the OM [32]. Plasminogen, in the complex with CipA, is cleaved and converted to the active serine protease plasmin, which degrades fibrinogen and the complement component C3b. Thus, CipA can prevent entrapment of *A. baumannii* in fibrin thrombi, and it facilitates dissemination of the pathogen. Regardless of plasminogen binding, CipA can also inhibit the alternative complement pathway [32]. The PKF serine protease is involved in serum resistance possibly through degradation of yet unidentified complement components [74]. It was suggested that both CipA and PKF are secreted via a type II secretion system (T2SS) [75]. The T2SS enables secretion of effector proteins including multiple enzymes critical for *A. baumannii* virulence [9]. The hypothesis that the T2SS participates in the secretion of CipA and PKF is based on results showing that deletion of the T2SS gene *gspD* resulted in significantly decreased resistance to human serum [75].

Recent studies revealed that, in addition to the aforementioned proteins and structures, *A. baumannii* possesses numerous protective mechanisms against complement-mediated killing. Sanchez-Larrayoz et al. identified 50 genes essential for the survival of *A. baumannii* in human serum, including the Mla system, which encodes proteins required for the maintenance of OM lipid asymmetry [54]. The Mla proteins prevent the accumulation of phospholipids in the outer leaflet of the OM by transporting them to the inner membrane. This study suggests that the accumulation of surface-exposed phospholipids in *mla*-deficient strains can activate the alternative pathway of the complement system [54].

An example of a metabolic adaptation that enables *A. baumannii* to evade neutrophil chemotaxis is the phenylacetic acid catabolism pathway encoded by the *paa* system [14]. The *paa* genes were identified in 16% of the sequenced bacterial species [76,77]. The *paa* operon is involved in degradation of aromatic compounds including phenylacetate to form acetyl-coenzyme A (CoA) and succinyl-CoA [76]. Bhuiyan et al. demonstrated that the loss of function of this catabolic pathway resulted in the accumulation of phenylacetate, which acted as an attractant of neutrophils, leading finally to bacterial clearance [14].

An interesting example of the *A. baummanii* virulence strategy is its ability to adhere to neutrophils without being eliminated by phagocytosis. Since neutrophils can transmigrate from the infection site to vasculature, it was proposed that the reverse migration of neutrophils can disseminate the infection to other organs [17]. Moreover, Sato et al. demonstrated that MDR *A. baumannii* isolates can survive in macrophages after phagocytosis. The MDR strains induced ROS production in macrophages, and they concomitantly exhibited upregulated catalase activity which allowed them to resist oxidative stress [78]. These results indicate that *A. baumannii* can spread in the infected organism using both neutrophils and macrophages.

**Figure 1:**
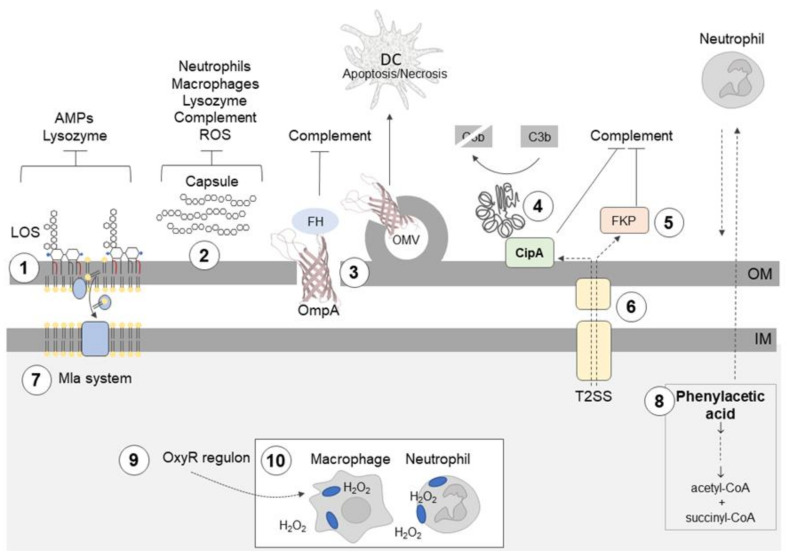
*Acinetobacter baumannii* uses different mechanisms to evade the innate immune response. **①** Hepta-acylation of lipid A in lipooligosaccharide (LOS) fortifies the outer membrane (OM) and protects *A. baumannii* from cationic antimicrobial peptides (AMPs), colistin, and lysozyme. **②** Highly hydrophilic and negatively charged capsular polysaccharides (CPS) hinder interactions with negatively charged surfaces of neutrophils and macrophages; the capsule is also a barrier which protects against complement-mediated killing, lysozyme degradation, and reactive oxygen species (ROS). **③** Outer membrane protein A (OmpA) interacts with factor H (FH), thereby inhibiting the complement-mediated killing; OmpA induces ROS production and the death of dendritic cells (DCs). **④** CipA forms a complex with plasminogen/plasmin, which degrades the complement component C3b; CipA and **⑤** the protein killing factor (PKF) serine protease inhibit the alternative complement pathway. **⑥** The type II secretion system (T2SS) contributes to serum resistance, and it probably participates in CipA and PKF serine protease secretion. **⑦** Surface-exposed phospholipids are potential activators of the alternative complement pathway. The Mla system prevents the accumulation of phospholipids in the outer leaflet of the OM. **⑧** Phenylacetate, a derivative of phenylalanine and neutrophil attractant, is removed from the bacterial cell by conversion to acetyl-coenzyme A (CoA) and succinyl-CoA. **⑨** Enhanced catalase activity enables *A. baumannii* to survive in macrophages in the presence of ROS. **⑩** *A. baumannii* can spread during infection using neutrophils and macrophages.

Most of the strategies described above (Figure 1) allow *A. baumannii* not only to evade the innate immune response, but also to survive in the external environment.

## 3. Mechanisms Protecting *A. baumannii* against Desiccation

Desiccation, as a common environmental stressor, poses challenges to bacterial cells. Water molecules, as the only nonvolatile solvent in cells, are critical in reaction mechanisms; they also confer stability to lipids, DNA, and proteins, as well as contributing structural order. Loss of membrane integrity during desiccation disrupts the respiratory chain leading to the accumulation of superoxide ions. Furthermore, the malfunction of transport proteins and destabilization of proteins with iron-sulfur clusters cause an increase in the level of intracellular iron. Superoxide radicals can participate in Fenton and Haber-Weiss reactions with ferrous or ferric ions leading to the production of highly toxic hydroxyl radicals [79]. Therefore, oxidative damage of DNA, lipids, and proteins is one of the effects of water loss. *Acinetobacter* spp., compared with other Gram-negative rods, are more resistant to dry conditions [80]. *A. baumannii* uniquely survives on inanimate objects and fingertips for extended periods, which explains its potency in cross-infection breakouts [81].

The main structures that facilitate bacteria to enhance water retention are CPS and LPS/LOS [36]. CPS were shown to contribute to desiccation tolerance in *A. baumannii* [51]. However, other studies demonstrated that there is no simple correlation between the capsule thickness and survival rate under desiccation [49]. Therefore, it seems that the type and structure of CPS or other mechanisms, including hepta-acetylated lipid A, must contribute to the outstanding desiccation tolerance of *A. baumannii*. Boll et al. demonstrated that the *A. baumannii* mutant lacking the LpxM acylsynthetaze produces penta-acylated lipid A, instead of hepta-acylated lipid A, and it exhibits decreased desiccation tolerance, probably due to increased membrane fluidity [59].

The accumulation of organic osmolytes, generally designated as compatible solutes, is a prerequisite for the adaptation of bacteria to osmotic stress imposed by water loss. A crucial role in desiccation resistance in various microorganisms is played by the non-reducing disaccharide, trehalose. Trehalose acts as an osmolyte, chemical chaperone, and metabolite that can directly or indirectly stabilize proteins and membranes [82,83,84,85]. It seems that endogenous trehalose is not involved in desiccation tolerance in *A. baumannii,* but exogenous trehalose was found to efficiently protect *A. baumannii* on dry surfaces [68]. In response to osmotic stress, *A. baumannii* also accumulates mannitol and glutamate; however, their contribution to desiccation resistance remains mostly unexplored [81]. To counteract the effects of oxidative stress, the expression of anti-oxidant enzymes, such as catalases KatE and KatG, superoxide dismutase, and glutathione peroxidase, is induced in desiccation-stressed *A. baumannii* [49,86,87]. A recent study by Farrow et al. proved that the global BfmR regulator contributes to that desiccation tolerance [49].

Gayoso et al. found that desiccation stress affects the composition of the OM. The overproduction of OMPs (Omp25, DcaP-like, and CarO) was observed, indicating a shift in membrane permeability. This study also revealed that several genes encoding proteins involved in transcription and translation, including RNA polymerase subunits RpoA and RpoC, ribosome-associated proteins, and the elongation factor Tu, are downregulated in *A. baumannii* during desiccation. Proteins whose expression was upregulated included ribosomal recycling factor (RRF), integration host factor (IHF), and the histone-like protein HU. RRF facilitates disassembly of the ribosome at the end of translation. IHF and HU are involved in transcription regulation, and they are essential for maintaining DNA supercoiling and compaction. Consistent with this finding, the presence of an electron-dense region inside desiccation-stressed *A. baumannii* cells was detected [87]. All these observations led to the conclusion that *A. baumannii* cells exposed to desiccation stress enter a dormant state [87]. Under favourable conditions, dormant bacteria can recover and resume growth.

The protection of proteins is crucial for the survival of bacteria during desiccation stress and subsequent rehydration. In the next section, we discuss mechanisms that counteract protein damage caused by water loss and other stresses. Then, we present the current knowledge of biofilm formation, which is one of the main strategies used by bacterial populations to survive desiccation stress [88].

## 4. Protein Homeostasis in *A. baumannii*

Upon oxidative stress, proteins, which are the main target of ROS, are damaged by metal-catalyzed oxidation and non-enzymatic glycation [79,82,89]. During desiccation, these detrimental reactions are facilitated by the reduction of the hydration shell around proteins and protein condensation, which in turn may lead to misfolding and aggregation of proteins. Apart from anti-oxidant enzymes, bacteria evolved additional mechanisms that protect proteins, including molecular chaperones and proteases. The main role of molecular chaperones is maintaining protein homeostasis (proteostasis), i.e., a proper balance of protein synthesis, folding, transport, and degradation [90]. Molecular chaperones are highly conserved among prokaryotes and, under stress conditions, they prevent aggregation of unfolded proteins, facilitate degradation of irreversibly misfolded proteins by proteases, and enable solubilization of protein aggregates for subsequent refolding or degradation [91,92]. The key molecular chaperones in bacteria include the heat-shock protein 70 (Hsp70) family chaperone DnaK, its DnaJ (Hsp40) co-chaperone, and the nucleotide exchange factor GrpE, as well as the chaperonin GroEL (Hsp60) and its co-chaperone GroES (Hsp10). The efficient solubilization of aggregated proteins requires the cooperation of the DnaK–DnaJ–GrpE system with ClpB (Hsp100), IbpA/B (the small Hsp family), or Hsp33, which is the primary chaperone redox-activated upon oxidative stress. Most of these chaperones were found to be upregulated in *A. baumannii* submitted to stresses that impair homeostasis: heat shock (DnaK, GroEL), oxidative stress (GrpE, DnaK, GroES, GroEL), antibiotic exposure (DnaK, GroEL), and desiccation (TF, GroES, GrpE, DnaJ, DnaK, ClpX, ClpB) [18,86,87,93,94]. Wang et al. reported that the expression of more than 50 genes encoding proteins related to proteostasis, including chaperones and the Lon protease, was increased during desiccation [86]. The induction of proteins involved in the proteostasis system was accompanied by protein aggregation. Surprisingly, the accumulation of protein aggregates correlated positively with the ability of *A. baumannii* to survive desiccation. The survival rate was also increased when protein aggregation was induced prior to desiccation by a subinhibitory concentration of streptomycin, or it was enhanced by the *Δlon* mutation. It was also demonstrated that the model proteins sequestered in the aggregates, β-lactamase and GFP, retained their activities [86]. These results are in agreement with previous studies showing that bacterial inclusion bodies contain functional proteins, and they confirm that aggregates may serve as compartments that protect proteins against inactivation [95,96]. Upon desiccation, the sequestration of native molecules into aggregates may be favored due to the gradual concentration of proteins. In contrast, high temperatures or other stressors that cause fast and abundant protein misfolding may lead to decreased survival or cell death due to the formation of aggregates enriched in non-functional proteins.

Recent studies showed that the induction of protein aggregation and disturbance of proteostasis may be an efficient strategy to kill pathogenic bacteria. Khodaparast et al. identified several peptides that induced bactericidal protein aggregation in *Escherichia coli* and *A. baumannii* [97]. The peptides contained aggregation-prone sequences (APRs) that naturally occur in hydrophobic cores of globular proteins or on protein–protein interaction surfaces. When aggregation was nucleated in bacteria by the peptides containing APRs, it led to the lethal formation of inclusion bodies containing hundreds of proteins. The quaternary amine compounds (QACs), including benzalkonium chloride (BZK), can also trigger protein aggregation in *A. baumannii* when used at low concentrations [98]. QACs are commonly used biocides that, at high concentrations, disrupt membranes. The exact mechanism of BZK action on proteostasis remains unclear, although it was found that resistance to BZK was acquired through ribosomal protein mutations that protected *A. baumannii* against BZK-induced protein aggregation.

## 5. Biofilm and Heterogeneity of *A. baumannii* Populations

### 5.1. Formation of A. baumannii Biofilms

**Figure 2:**
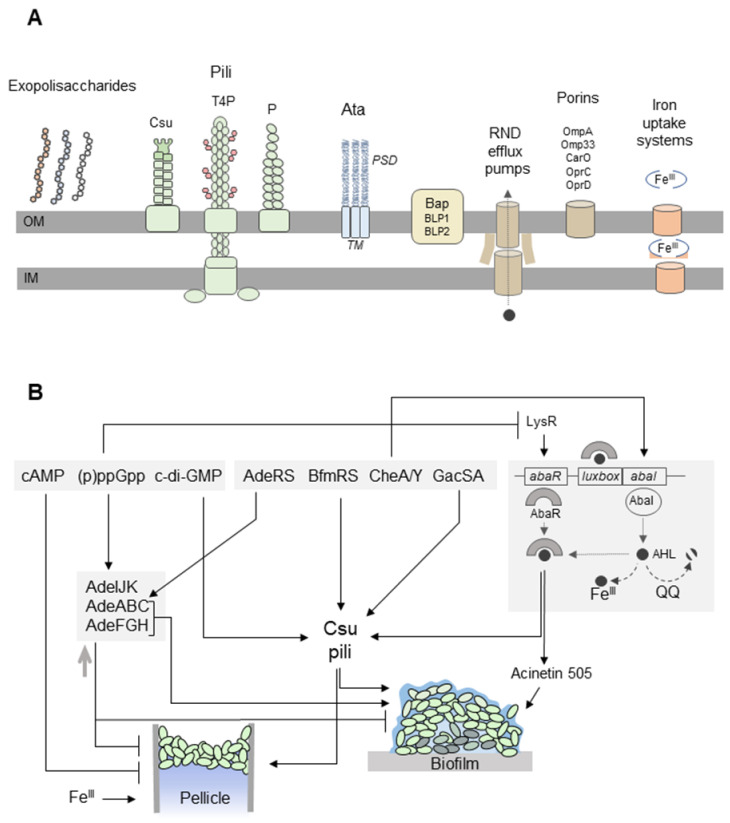
*A. baumannii* forms biofilms on solid surfaces and pellicles at the air-liquid interface. (**A**) Extracellular appendages involved in biofilm/pellicle formation include exopolysaccharides (capsular polysaccharides, poly-β-(1–6)-*N*-acetylglucosamine (PNAG), alginate), and pili [108]. Csu pili are assembled via the chaperone-usher pathway. The CsuE adhesin, which is located at the pilus tip, exposes three hydrophobic finger-like loops that may insert into cavities in abiotic surfaces [109]. Type IV pili (T4P) are composed of PilA subunits with variable amino-acid sequences in different *A. baumannii* strains. Depending on the PilA structure, the pili promote twitching motility or biofilm formation [110]. The chaperone–usher P pili are overproduced in *A. baumannii* pellicles [105]. The homotrimeric Ata autotransporter binds to extracellular matrix components and abiotic surfaces. The transmembrane anchor domain (TM) facilitates the export of the passenger domain (PSD) to the cell surface through a pore formed by the TM. Flexible PSDs allow interactions with different surfaces [111]. Bap and Bap-like proteins (BLP1, BLP2) stabilize the three-dimensional biofilm structure on abiotic surfaces and play a role in the adhesion of *A. baumannii* to the host cell [112,113]. Three resistance-nodulation-division (RND) efflux pumps (AdeABC, AdeFGH, and AdeIJK) affect *A. baumannii* biofilm development [114,115,116]. The AdeFGH efflux pump participates in the transport of quorum-sensing (QS) molecules [116]. OmpA is responsible for the attachment of *A. baumannii* to plastic surfaces and epithelial cells [67]. The CarO, OprC, and OprD porins may be involved in the uptake of metabolites required for the synthesis of siderophores in pellicles [105]. The iron uptake systems, including acinetobactin and enterobactin receptors, are upregulated during pellicle formation [105,117]. (**B**) The formation of the *A. baumannii* biofilm and pellicle is regulated by the nucleotide second messengers, two-component signal transduction systems, and QS. cAMP inhibits pellicle formation [104]. The synthesis of Csu pili depends on cyclic di-GMP (c-di-GMP) [118] and the BfmRS and GacSA systems [48,119,120]. The hybrid two-component regulator CheA/Y controls the expression of Csu pili and acinetin-505 via QS [106,121]. The QS system of *A. baumannii* consists of an AbaI inducer and its cognate receptor AbaR. AbaI is an autoinducer synthase producing *N*-acyl homoserine lactone (AHL) molecules bound by AbaR. The AbaR–AHL complexes activate the synthesis of AbaI and the expression of QS-dependent genes, which in turn triggers the production of acinetin-505 and Csu pili [121,122]. Biofilm formation may be inhibited by quorum-quenching (QQ) enzymes, which degrade AHLs [123,124], as well as high concentrations of Fe^III^ ions that bind AHLs [125]. On the other hand, Fe^III^ ions are required for pellicle development [105,117]. The AdeABC (controlled by the two-component signal transduction AdeRS system) and AdeFGH efflux pumps participate in biofilm formation [114,116]. However, other studies revealed that the overproduction of efflux pumps may result in decreased biofilm/pellicle growth [115]. ppGpp regulates the expression of genes encoding the efflux pump’s components [126] and inhibits the production of AbaR and acinetin-505 [127].

Biofilms are multicellular consortia of single or multiple bacterial species enclosed in extracellular polymeric substances (EPS) which comprise polysaccharides, proteins, and nucleic acids secreted by bacteria. The structure of mature biofilms is often very complex with clusters of bacterial cells separated by fluid-filled channels. Diffusion of nutrients and oxygen is limited in biofilms; therefore, the environmental conditions are not homogeneous throughout a biofilm. This leads to the formation of heterogeneous cell subpopulations adapted to local microenvironments. Biofilm-dwelling bacteria are more resistant to antibiotics and other stressors than planktonic cells [99,100]. A number of factors are known to lead to the enhanced antibiotic resistance of biofilms, e.g., impaired drug diffusion due to microbial aggregations and overexpression of the extracellular polymeric substance (EPS) matrix, biofilm-specific efflux pumps, alterations in microbial phenotypic and genotypic features due to stress responses, and specific microenvironment conditions that inactivate antibiotics and the presence of persister cells (see below) [101]. Antibiotics administered at concentrations below the minimum inhibitory concentration (MIC) often induce biofilm formation in a variety of bacterial species [100,102]. *A. baumannii* forms biofilms on both biotic and abiotic surfaces which contributes to its remarkable ability to survive in hospital environments. While extrinsic factors such as surface hydrophobicity, temperature, and oxygen concentration are reported to influence *A. baumannii* biofilms, numerous physicochemical and microbial features (e.g., capsular polysaccharides, surface appendages, adhesins, and virulence and resistance determinants) facilitate the formation and maintenance of *A. baumannii* biofilms (Figure 2A) [103]. In addition to biofilms on solid surfaces, *A. baumannii* also forms “pellicles” at the air-liquid interface (Figure 2B) [104,105]. The formation of these floating biofilms is a rare trait in clinical *A. baumannii* isolates, and it is associated with surface-associated motility [104,106]. The relationship between motility and pellicles or surface-attached biofilms is complex. Although a motile state seems to be the opposite to a sedentary lifestyle in biofilms, motility may be required for the formation of microcolonies at the early stages of biofilm development and during the reorganisation of mature three-dimensional biofilm structures [107].

*A. baumannii* does not produce flagella; however, it can move via surface-associated motility or twitching motility [104,128]. Multiple genes required for surface-associated motility, including genes associated with purine and pyrimidine biosynthesis or natural competence, were recently identified [128], but its mechanism remains poorly understood. Twitching motility is mediated by the extension and retraction of type IV pili, which are composed of helically assembled PilA subunits. PilA produced by various *A. baumannii* isolates differ in amino-acid sequence and *O*-linked glycosylation. It was proposed that, when negatively charged residues dominate on the surface of the headgroup domain of PilA, the pili retract from each other due to electrostatic repulsion and promote twitching motility. The opposite effect, i.e., pili bundling, cell–cell attachment, and biofilm formation, may occur in the case of PilA variants without negatively charged headgroup domains [110].

Several studies showed that one of the main structures required for cell attachment and biofilm development is CPS [129]. For example, it was shown that, in the case of the *A. baumannii Δwza*, capsule-deficient strain biofilm growth and adhesion to epithelial cells were reduced [45]. However, under certain conditions, enhanced production of the capsule may be associated with biofilm reduction. It was recently reported that the Lon protease affects biofilm formation in *A. baumannii* [130]. Although the Lon-deficient mutant produced thicker capsule compared to wild-type (WT) cells, it displayed lowered adherence to polystyrene surfaces, decreased motility, and formed a weak pellicle biofilm, but strongly upregulated a surface antigen, encoded by *surA1*. The exact mechanism of biofilm regulation by Lon remains to be elucidated. *A. baumannii* produces an additional surface exopolysaccharide, poly-β-(1–6)-*N*-acetylglucosamine (PNAG). Proteins involved in the polymerization and secretion of PNAG are encoded by the *pgaABCD* operon widely distributed among *A. baumannii* clinical isolates [131].

The ability of *A. baumannii* to form biofilms on abiotic surfaces depends on the production of pili assembled via the CsuA/BABCDE chaperone–usher secretion system which is controlled by the BfmR global regulator [109,119]. OmpA also participates in the development of biofilms on plastic surfaces. In contrast to the CsuA/BABCDE pili system, OmpA is required during the attachment to *Candida albicans* filaments and human alveolar epithelial cells. After the attachment, OmpA triggers apoptosis of the eukaryotic cells [67]. The giant Bap protein, consisting of 8621 amino acids, is involved in the formation and stabilization of the complex three-dimensional biofilm architecture on abiotic surfaces, and it plays a role in adhesion of *A. baumannii* to the host cell [112,113]. Bap possess immunoglobulin-like (Ig-like) repeats that seem to be typical for proteins involved in biofilm development, for example, Bap-like proteins, BLP1 and BLP2, produced by some *A. baumannii* strains [112,132]. Another surface adhesin in *A. baumannii*, the trimeric Ata autotransporter, is involved in biofilm production by binding various extracellular matrix/basal membrane (ECM/BM) components, including the basement protein laminin and collagen types I, III, IV, and V. During tissue damage, ECM/BM proteins become exposed serving as docking sites for *A. baumannii* and a niche supporting biofilm growth. Ata is also responsible for self-adhesion of *A. baumannii* cells and biofilm formation on various abiotic materials [111,133,134]. Other proteins and structures contributing to *A. baumannii* biofilm development comprise CarO, Omp33, the resistance–nodulation–division (RND) efflux pumps, Pap pilus, and alginate [102,103,108,114,116,135,136]. Depending on the experimental conditions, various structures and mechanisms responsible for pellicle formation were identified [104,105,106,117,137]. Two independent studies demonstrated that the iron uptake systems, as well as CarO, OprD, and OprC porins, are required to develop pellicles [105,117] In addition, the overexpression of multiple pili systems, including Fil and Csu pili, was also observed [105,117,137].

At least three two-component systems regulate surface motility and biofilm/pellicle formation: the aforementioned BmfRS pathway, GacSA, and CheA/Y [48,106,120]. CheA/Y is a hybrid sensor histidine kinase/response regulator that controls the *csuA*/*ABCDE* operon and the AbaI-dependent quorum-sensing (QS) pathway [106]. The QS system of *A. baumannii* consists of AbaI autoinducer synthase and the AbaR receptor protein for the autoinducer, *N*-acyl homoserine lactone (AHL) (for more details, see Figure 2B) [138]. Different types of AHLs were detected in *A. baumannii* [139,140,141]. Interestingly, C8-AHL and 3-oxo-C8-AHL were produced by both soil and nosocomial *A. baumannii* strains, whereas long-chain AHLs with C10, C12, C14, and C16 acyl chains were detected only in the nosocomial isolates [140]. During biofilm formation, the AdeFGH efflux pump participates in the transport of AHLs [116]. It was suggested that QS signals may initiate twitching motility and the attachment of *A. baumannii* to abiotic surfaces via the CsuA/BABCDE secretion system [122]. Other studies reported that AbaI is required for the later stages of biofilm development [139,141]. Since QS signaling molecules in some bacteria are strong iron chelators, ferric iron (Fe^3+^) limitation increases the AHL level in *A. baumannii* in a dose-dependent manner, leading to a stress response and biofilm formation [125].

Nucleotide second messengers such as cAMP, cyclic di-GMP (c-di-GMP), and penta/tetra-guanosine phosphate ((p)ppGpp) are key regulators of numerous bacterial traits including adaptation to harsh environments, as well as transition from biofilm to motility, mutualism to commensalism, acute to chronic virulence characteristics, and cell division to cell differentiation [142,143]. The exact functions of these messengers in *A. baumannii* were only partially examined. It was reported that enhanced cAMP levels, caused by the lack of cAMP phosphodiesterase, lead to the inhibition of surface-associated motility and pellicle formation [104]. C-di-GMP is synthesized by diguanylate cyclase activity of GGDEF domain-containing proteins, while degradation of c-di-GMP into two GMP molecules is catalyzed by the phosphodiesterase activity of EAL domain-containing proteins [144,145]. Most c-di-GMP-dependent signalling pathways regulate the bacteria ability to interact with abiotic surfaces or with other bacterial and eukaryotic cells. Eleven genes for GGDEF/EAL proteins in the genome of the *A. baumannii* 17*,*978 strain were identified, and most of the predicted proteins were enzymatically active [118]. It was demonstrated that distinct panels of these enzymes promote biofilm formation, macro-colony growth, and surface-associated motility [118].

The (p)ppGpp alarmone is produced by the RelA/SpoT proteins in response to amino-acid starvation and other stresses. (p)ppGpp triggers the stringent response resulting in the downregulated transcription of most metabolic genes and the upregulation of genes responsible for amino-acid biosynthesis [146,147,148]. The stringent response was linked to biofilm formation in a range of pathogens, including *Acinetobacter* spp. [142,149,150]. The formation of biofilms is impaired [149,151] or enhanced [152,153] in (p)ppGpp-deficient bacteria. Recent studies revealed the interplay among the stringent response, QS, motility, and biofilm/pellicle formation in *A. baumannii*, but the exact mechanisms remain unclear (Figure 2B). It was reported that the formation of *A. baumannii* biofilms can be inhibited by a synthetic dodecapeptide 1081, which triggers degradation of (p)ppGpp [149]. However, the activity of peptide 1081 and its link with the stringent response were recently questioned [154]. The Δ*relA* mutation in *A. baumannii* results in a hypermotile phenotype, as well as in the overproduction of AbaR and acinetin-505. Acinetin-505 is a 505-Da lipopeptide that may act as a surfactant promoting surface-associated motility [127], biofilm formation, and virulence [121]. Numerous studies indicated that (p)ppGpp is involved in the formation of dormant persister bacteria, which are implicated in biofilm tolerance to antibiotics [127,155].

### 5.2. Persisters and Heterogeneity of A. baumannii Populations

Persisters are able to survive exposure to a bactericidal drug concentration, and they usually constitute a small fraction of bacterial populations [156]. Antibiotic persistence is a transient state, and, when persisters resume growth after drug treatment, their progeny become antibiotic susceptible. There is an ongoing debate about mechanisms underlying persister formation [157,158,159,160]. It is well known that biofilms provide conducive niches that favor the formation of persisters [161]. Persisters can arise spontaneously or in response to stress caused by antibiotics, the host immune response, ROS, high osmolarity, pH changes, diauxic shift, desiccation, or nutrient starvation. In addition to the stringent response mentioned above, toxin–antitoxin modules, quorum signaling, efflux pumps, the SOS, and oxidative stress responses can be activated during persister formation. These pathways and stimuli may lead to decreased metabolism, depletion of ATP, protein aggregation, and inhibition of translation [132,162,163]. It was recently demonstrated that, in *E. coli*, (p)ppGpp induces production of the ribosome modulation factor (RMF), the hibernation-promoting factor (Hpf), and the ribosome-associated inhibitor (RaiA), which convert active 70S ribosomes into inactive 70S, 90S, and 100S ribosomes, leading to translation inhibition [164]. It should be noted that, due to diverse conditions in biofilm structures, multiple mechanisms triggering antibiotic tolerance, and stochastic effects, persister subpopulations should be considered as a heterogeneous group of cells.

The formation of *A. baumannii* persisters induced by polymixin B, meropenem, and ceftazidime was reported [165,166,167,168,169]. It was also found that the ppGpp deficiency in the *A. baumannii ΔrelA* strain reduced formation of persister cells tolerant to colistin and rifampicin [127]. The analysis of the transcriptome of persisters tolerant to ceftazidime revealed upregulation of two toxin–antitoxin systems HigB/HigA and DUF1044/RelB, as well as downregulation of certain metabolic pathways, including the electron transport chain and citrate cycle [168]. Interestingly, the expression of genes associated with biodegradation pathways of aromatic compounds was detected in persistent cells. It was suggested that the degradation of aromatic rings in antibiotics could be utilized by *A. baumannii* persisters during nutrient starvation [168]. Zou et al. found that the major fraction of *A. baumannii* persisters that survive β-lactam antibiotic treatment contains spherical non-walled, but metabolically active cells [170]. In contrast to wall-deficient, so-called L-forms of other Gram-negative bacteria, *A. baumannii* non-walled cells were able to survive without any osmoprotective agent. This type of *A. baumannii* persister cell was also formed during antibiotic therapy in vivo in *Galleria melonella* larvae which were used as the infection model.

Biofilms provide a conducive environment facilitating not only phenotypic heterogeneity (e.g., persister formation) but also genetic diversification. It was demonstrated that evolution within *A. baumannii* biofilms can generate greater genetic diversity than in planktonic, well-mixed populations [171]. Planktonic cells exposed to ciprofloxacin shared the same limited number of mutations in topoisomerase (the primary drug target), whereas biofilm-adapted populations acquired different types of mutations in the regulators of the efflux pumps. The emergence of a certain trade-off between fitness and resistance level was detected; biofilm-adapted clones were less drug-resistant than planktonic cells, but more fit in the absence of the drug [171]. Other studies demonstrated that the exposure of *A. baumannii* biofilms to sub-inhibitory concentrations of ciprofloxacin or tetracycline led to the generation of genetic and phenotypic diversity among biofilm dispersal isolates [172]. Dispersed cells accumulate a wide diversity of mutations that enhance biofilm formation and antibiotic resistance. For example, the efflux transport system AdeABC was upregulated in the presence of both ciprofloxacin and tetracycline, whereas the expression of RecA and UmuD, which are involved in DNA repair and mutagenesis, was increased during ciprofloxacin treatment.

Phenotypic alteration between opaque (VIR-O) and translucent (AV-T) colonies is another example of *A. baumannii* population heterogeneity. It was shown that both phenotypes exhibited significant differences in cell morphology, biofilm formation, surface motility, antibiotic resistance, and virulence [173]. VIR-O cells were covered with a thicker coating of the extracellular capsule, and they were more resistant to disinfectants, ROS, antibiotics, lysozyme, and the cathelin-related antimicrobial peptide [53]. This highly virulent subpopulation dominated in the bloodstream of human patients. The AV-T cells produced more dense biofilms and a larger quantity of OMVs in comparison with the VIR-O variant [174]. The analysis of VIR-O and AV-T transcriptomes suggested that the AV-T subpopulation is better adapted for natural environments outside the host than VIR-O cells. The phenotype switching between VIR-O and AV-T subpopulations depends on several factors, including a TetR-type transcriptional regulator ABUW_1645, the ArpAB efflux system, the EnvZ/OmpR two-component system [174], and ppGpp [127].

Antibiotic heteroresistance is another common phenotype that may contribute to the heterogeneity of bacterial populations. We describe this phenomenon in the next section, discussing the broader problem of multidrug resistance *of A. baumannii*.

## 6. Multidrug Resistance of *A. baumannii*

Multidrug-resistant pathogens pose serious threats in healthcare settings worldwide. For the past number of years, antimicrobial discovery and resistance development to new antimicrobials occurred almost at the same time. Not surprisingly, *A. baumannii*, similarly to other bacteria, also acquired resistance to newly developed antimicrobial agents [175]. To characterize the various patterns of resistance, the following terms are used: MDR, extensively drug-resistant (XDR), and pandrug-resistant (PDR) bacteria. According to the definition proposed by Magiorakos et al. [176], MDR is defined as acquired non-susceptibility to at least one agent in three or more antimicrobial categories, XDR is defined as non-susceptibility to at least one agent in all but two or fewer antimicrobial categories, and PDR is defined as non-susceptibility to all agents in all antimicrobial categories. In the case of *Acinetobacter* spp., 22 antimicrobial agents belong to nine categories: aminoglycosides, antipseudomonal carbapenems, antipseudomonal fluoroquinolones, (antipseudomonal) penicillins + β lactamase inhibitors, extended spectrum cephalosporins, folate pathway inhibitors, polymixins, and tetracyclines. *A. baumannii* can become resistant to a variety of antibiotics via intrinsic and acquisition mechanisms. Its ability to acquire drug resistance genes from other human pathogens is not well understood. However, considering the capability of the *A. baumannii* genome to exchange genetic material both within and between species, it is quite likely that these bacteria may have evolved toward enhanced pathogenicity.

### 6.1. Mechanisms Responsible for A. baumannii Multidrug Resistance

The main mechanisms conferring resistance to different classes of antibiotics include the presence of β-lactamases, modifying enzymes, permeability defects, alteration of target sites, and multidrug efflux pumps [177]. Severe hospital-acquired infections caused by *A. baumannii* involve the use of carbapenems as highly effective drugs of choice used for the treatment of such infections [178]. Because of their broad spectrum, carbapenems are often active against microorganisms resistant to other antimicrobial compounds, and they are frequently used to treat complicated bacterial infections. Over the last few years, *A. baumannii* MDR strains became increasingly resistant to carbapenems, the drug of choice to treat severe infections caused by these bacteria. The main cause of carbapenem resistance is the presence of oxacillinases (OXA), which belong to the Ambler class D β-lactamases. Over 400 OXA enzymes encoded by chromosome- or plasmid-located genes were characterized. Other classes of β-lactamases: class A, class B (metallo-β-lactamases, MBL), and class C (AmpC) were also identified in *A. baumannii* strains [6,179,180,181,182,183,184,185]. The most frequent MBLs in *A. baumannii* are imipenemases (IMPs), Verona integron-encoded MBL (VIM), and MBL from New Delhi (NDM). Class C β-lactamases are encoded by the *ampC* gene. Overexpression of *ampC*, regulated by an upstream insertion sequence (IS) element known as IS*Aba1*, is the main mechanism of resistance to third-generation cephalosporins in *A. baumannii* [186]. Overexpression of the OXA and AmpC enzymes due to the presence of IS elements (see below), as well as the emergence of new OXA and AmpC variants, contributes to the increasing problem of *A. baumannii* resistance [187,188].

Another mechanism of *A. baumannii* resistance is associated with enzymatic modification of the antimicrobial molecule. One of the best examples of resistance via modification of the drug is the presence of a large group of aminoglycoside-modifying enzymes (AMEs). These enzymes possess unique substrate specificity and modify amino- or hydroxyl- groups of the aminoglycosides. There are three different types of AMEs, acetyltransferases, nucleotidyl transferases, and phosphotransferases, while all of them were identified in *A. baumannii* isolates [6].

Proteomic analysis of *A. baumannii* MDR strains shows protein variability that could be correlated with the appearance of resistance phenotypes, especially OMPs, which are involved in cellular drug uptake or efflux. The emergence of an antibiotic resistance level is often related to diverse variations in the expression of OMPs. It was found that, in *A. baumannii*, OmpA is strongly associated not only with adhesion to epithelial cells and biofilm formation, as mentioned earlier, but also with the modulation of cellular permeability and antibiotic resistance [103]. Importantly, changes in permeability frequently result in low-level resistance; therefore, the combination with other mechanisms, such as increased expression of efflux pumps, to confer a high-level antibiotic resistance phenotype is required [189].

Another common mechanism of antibiotic resistance in *A. baumannii* is alteration of the target site or cellular functions [2]. This often results from spontaneous mutation of a bacterial gene on the chromosome. Modification of the target site results in decreased affinity for the drug molecule. One of the most known examples of target changes is enzymatic alteration of the binding site. In an alternative pathway, bacteria produce new proteins that protect the target against an antibiotic. Examples of drugs affected by this mechanism include fluoroquinolones and tetracyclines [190]. In *A. baumannii*, point mutations in the *gyrA/parC* topoisomerases result in fluoroquinolones resistance, whereas a mutation in *rpsJ*, the gene that encodes the ribosomal S10 protein, is responsible for tigecycline resistance [191]. Another interesting example is the mechanism responsible for colistin resistance. Positively charged colistin kills bacteria by interacting with the negatively charged lipid A and destabilization of the OM. Mutations in the lipid A biosynthesis genes, *lpxA*, *lpxC*, and *lpxD*, result in the complete loss of lipooligosaccharides, which in turn abolish interactions with colistin. The second mechanism depends on the PmrAB two-component system. Mutations in *pmrA* or *pmrB* lead to the activation of the *pmrC* gene located upstream of *pmrAB,* encoding phosphoetanolamine transferase. Phosphoethanolamine transferred to lipid A decreases the negative charge of LOS, preventing colistin binding [62,63].

Among several types of efflux pumps that confer multidrug resistance*,* the RND efflux systems (AdeABC, AdeFGH, AdeIJK) are the most prevalent in *A. baumannii*. The AdeABC pump, found in 80% of *A. baumannii* isolates, extrudes a wide range of antibiotics, including β-lactams, aminoglicosides, fluoroquinolones, tetracyclines-tigecycline, macrolides–lincosamides, and chloramphenicol [103,177,192].The expression of AdeABC is tightly regulated by the AdeRS two-component system which was found to control almost 600 other genes [192,193]. Point mutations in the *adeR–adeS* genes or the presence of an IS*Aba1* insertion sequence upstream from the *adeABC* operon result in the overexpression of the AdeABC pump and multidrug resistance [194]. The AdeFGH pump, when overexpressed, confers enhanced resistance to fluoroquinolones, tetracycline–tigecycline, chloramphenicol, clindamycin, trimethoprim, sulfamethoxazole, sodium dodecyl sulfate, and dyes such as ethidium bromide, safranin O, and acridine orange [195]. It was found that overexpression of AdeFGH is caused by mutation in the *adeL* gene located upstream from the *adeFGH* operon, as well as the encoding of an a-Lys-type transcriptional regulator. The AdeIJK pump is produced in *A. baumannii* constitutively, and it is responsible for resistance to the same major drug classes as AdeABC, as well as antifolates and fusidic acid [115].

### 6.2. Genetic Elements Responsible for A. baumannii Multidrug Resistance

Members of the genus *Acinetobacter* quickly develop resistance to antimicrobial compounds. Antibiotic resistance genes can be disseminated through various mechanisms of horizontal gene transfer such as transformation, conjugation, and transduction. *A. baumannii* appears to use all the mechanisms; however, recent studies point to natural transformation as the mechanism playing an important role in the acquisition of the multidrug resistance phenotype [196]. In this process, bacteria appeared to be capable of uptake, integration, and functional expression of naked fragments of extracellular DNA from the environment. Bacteria could use transformation to avoid being targeted by antibiotics by accepting the genetic variation present in their neighborhood, including drug resistance genes [197]. Multidrug resistance of *A. baumannii* is mainly due to the horizontal acquisition of resistance genes, although recent studies showed that increased expression of chromosomal genes for the efflux system plays a major role in MDR [177].

Often, *A. baumannii* resistance to more than one class of antibiotics occurs when genes encoding resistance to antimicrobial agents are physically located in close proximity to each other on mobile genetic elements such as plasmids, transposons, and integrons. Plasmid profiling revealed the presence of multiple plasmids of varying molecular sizes in more than 80% of *Acinetobacter* isolates [198]. They constitute a reservoir of genes important not only for the dissemination of antibiotic resistance but also essential for bacteria adaptation and evolution. Recent analysis of 173 complete plasmid sequences from *A. baumannii* isolates originated from 17 countries revealed that 34.6% of the plasmids pose antibiotic resistance genes [199]. Bertini et al. constructed a classification system for the *A. baumannii* plasmids based on the sequence of replicase genes, and they identified 19 homology groups (GR1–GR19) [200]. Fourteen additional groups of plasmids were recently proposed [199,201,202]. GR6 was the most prevalent group detected in antibiotic-resistant *A*. *baumannii* isolates from Europe [203]. The GR6 plasmids harbor class D β-lactamase genes, including *bla*_OXA-23_, *bla*_OXA-58_, and *bla*_OXA-40_, aminoglycoside-resistant genes (*aph*(3’)-Via, *aadB*, *aadA2*, *strA*, *strB*, *aacA4, aph*(3’)-Via), and sulfonamide (*sul2*) and streptomycin (*strAB*) resistance genes [199,200,203].

IS and transposons (Tn) are able to move from one genomic location to another in the chromosome or plasmid DNA within a single cell [204]. IS may include a strong promoter that initiates the expression of a downstream gene, e.g., IS*Aba1* located upstream of *bla*_OXA-51_ genes or the *adeABC* operon leading to intrinsic *A. baumannii* carbapenem resistance or multidrug resistance, respectively [194,205]. A diverse range of composite Tn, which harbor antibiotic resistance genes flanked by IS, was identified in *A. baumannii* isolates. These transposons encode AmpC cephalosporinases, OXA carbapenemases, aminoglycosidases, and NDM or VIM metallo-carbapenemases [179,186,206,207,208,209,210]. For example, a chromosomally located Tn125-like transposon containing the *bla*_NDM-1_ gene was identified in NDM-1-producing *A. baumannii* from European countries [179]. The *bla*_OXA-23_ genes with adjacent IS*Aba1* were detected in globally disseminated transposons Tn2006 and Tn2008, as well as in Tn2009 from *A. baumannii* strains isolated in China [206,208]. The largest antibiotic resistance gene clusters in various *A. baumannii* isolates are resistance islands designated AbaR1–R30. These complex transposons are located in the chromosomal *comM* gene (encoding the ATP-ase), and they carry heavy-metal resistance determinants apart from antibiotic resistance genes [125,211]. Most AbaRs from *A. baumannii* strains of international clone I share a backbone transposon Tn*6019* interrupted by a large compound transposon that contains a variable-resistance region flanked by two copies of Tn*6018* [212]. AbaR1 contains genes conferring resistance to ampicillin (*veb1* and *oxa10*), sulfonamides (three copies of the *sul1* gene), streptomycin (two copies of *aadA1*; *strA, strB*), aminoglycoside (*aadB, aacC1, aphA1b, aacA*), chloramphenicol (*cmlA1, cmlA5, cmlA9*, and *catA1*), rifampin (*arr2*), trimethoprim (*dfrA1* and *dfrA10*), and tetracycline (*tetA-A* and *tetA-G*) [211].

Integrons are responsible for the dissemination of antibiotic resistance, especially among Gram-negative bacteria [213]. They can integrate into chromosomes or plasmid via site-specific recombination. These genetic elements are able to acquire, integrate, and express gene cassettes which can carry antibiotic resistance. Class 1 integrons are commonly found in *A. baumannii* and typically encode genes for aminoglycoside resistance, Ambler class A β-lactamases, metallo-beta-lactamases, and oxacillinases, as well resistance to antiseptics and sulfonamides [196]. They were also reported in clinical *A. baumannii* strains. Many reports showed that clinical *A. baumannii* strains carrying integrons were significantly more resistant to all tested antibiotics than strains lacking integrons [214]. It should be noted that some mobile genetic elements and resistance genes are disseminated worldwide (e.g., IS*AbaI, bla*_OXA-23_, *bla_OXA-51_*), whereas others are distributed locally across different countries or regions (e.g., integrons and class B carbapenemases are more frequently found in Asia) [215,216].

### 6.3. Cross-Resistance, Co-Regulatory Resistance, and Stress-Induced Resistance to Antibiotics in A. baumannii

Antibiotics can induce selective pressure on bacterial populations, leading to antimicrobial resistance through a mechanism called cross-resistance. This mechanism confers resistance to an entire class of antibiotic and is mainly achieved via multidrug efflux pumps. In *A. baumannii*, efflux pumps can be specific for a single substrate or can confer resistance to multiple antimicrobials by facilitating the extrusion of a broad range of compounds including antibiotics, heavy metals, and biocides from the bacterial cell [177]. Other studies demonstrated that treatment of *A. baumannii* infection with cationic microbial peptide colistin can induce not only increased resistance to antibiotics but also resistance to host cationic antimicrobials typically found at sites of inflammation. These findings showed that understanding the molecular basis of cross-resistance is important for the development of more effective therapeutic schemes [217].

Another mechanism involved in bacterial resistance is called co-regulatory resistance. This occurs when resistant genes to antimicrobial agents are controlled by regulatory proteins [218]. Commonality of target sites between different class of antibiotics leads to the selection of mutants and the emergence of cross-resistance, as well as the co-selection and persistence of antibiotic-resistant strains. The presence of class 1 integrons in *A. baumannii* strains confers several phenotypes, including resistance, to a broad range of antibiotic classes, in addition to heavy metals and biocides [213]. In integrons, antibiotic resistance genes are under the control of a single promoter. As a result, these genes are expressed in a coordinated manner.

Some microorganisms readily acquire antibiotic resistance mechanisms in response to environmental stresses. It was shown that different physiological conditions influenced antimicrobial susceptibility and porin expression in *A. baumannii*. For example, putative efflux transporters were induced by the physiological concentrations of NaCl, contributing to increased tolerance of *A. baumannii* to aminoglycosides, carbapenems, quinolones, and colistin. Moreover, the physiological level of some cations present within the host promotes the upregulation of genes coding for multidrug efflux pumps [219]. Such regulated changes in efflux pump expression may increase the ability of this pathogen to survive antibiotic challenge.

As mentioned, many clinical *A. baumannii* strains can survive drying for a prolonged period of time. However, when rehydration happens, DNA damage may occur such as various DNA lesions, cross-linking, base removal, and strand breaks. To repair some of the DNA damage, *A. baumannii* developed an inducible DNA damage response in which RecA plays a major regulatory role in mechanisms involved in stress survival [220]. The RecA protein is involved in DNA damage repair and, consequently, in cellular protection against stresses induced by DNA damaging agents, several classes of antibiotics, and oxidative agents. This response increases mutagenesis and is one of the mechanisms used by *A. baumannii* to acquire antibiotic resistance, particularly in hospitals under clinically relevant DNA-damaging conditions [221].

In another study, it was shown that *A. baumannii* cells pretreated at 45 °C for 30 min were better able to survive a subsequent streptomycin exposure than cells pretreated at 37 °C. This phenomenon may be explained by the synthesis of misfolded proteins, produced by the streptomycin-disrupted ribosome and inserted into the bacterial membrane. Treating *A. baumannii* cells with the aminoglycoside antibiotic streptomycin induces not only ribosomal mistranslation but also expression of the heat-shock proteins DnaK and GroEL, responsible for elimination of aberrant polypeptides, thereby reducing their toxicity to bacterial cells [222].

The emergence of antibiotic-resistant *A. baumannii* strains may be preceded by the formation of persister cells. The evolution of antibiotic resistance promoted by persistence or tolerance was observed in vitro or in patients in the case of other bacterial species [223,224,225,226,227]. Persisters, as non-dividing cells, can accumulate de novo mutations via mechanisms independent of DNA replication or via horizontal gene transfer.

### 6.4. Heteroresistance to Antibiotics in A. baumannii

The term “heteroresistance” is defined as the presence of subpopulations of cells that have a higher MIC than the dominant population [228,229]. In contrast to persisters which are dormant cells, heteroresistant subpopulations can proliferate in the presence of antibiotics.

*A. baumannii* heteroresistance to colistin in “colistin-susceptible” clinical isolates was described for the first time by Li et al. [230]. Colistin heteroresistance was caused by mutations and the insertional inactivation of the lipid A biosynthesis genes, leading to the complete loss of lipooligosacchrides [63,231]. Heteroresistance to carbapenems, aminoglycosides, and trimethoprim/sulfamethoxazole in *A. baumannii* was also reported [167,232,233]. For example, it was found that increased tobramycin resistance was an unstable phenotype that emerged due to the extensive RecA-dependent amplification of the *aadB* gene encoding an aminoglycoside adenylyltransferase. The *aadB* gene was carried on a plasmid, in the region containing four other resistance genes [233]. Gene amplification seems to be the main mechanism conferring heteroresistance in various pathogens. Analysis of the prevalence and mechanisms of heteroresistance in *A. baumannii, E. coli, Salmonella enterica*, *and Klebsiella pneumoniae* revealed that almost 28% of clinical isolates were heteroresistant to various antibiotics. The majority of heteroresistance cases were unstable, and they resulted from tandem amplification of resistance genes [234].

## 7. Concluding Remarks

*A. baumannii* is the primary species detected and isolated from hospital environments including intensive care units. The ease with which *A. baumannii* colonizes patients makes it problematic as these patients might transmit or become infected when the immune system is stressed. Most clinical *A. baumannii* isolates are naturally competent; thus, they can rapidly acquire resistance genes. The success of *A. baumannii* as a human pathogen is also associated with its outstanding ability to survive long-term desiccation in nosocomial environments. The formation of biofilms and antibiotic-tolerant persisters contributes to the heterogeneity of *A. baumannii* populations, facilitating their adaptation to fluctuating environments. It was proposed that, in response to desiccation stress, *A. baumannii* follows the “bust-and-boom” strategy [87,235]. The “bust-and-boom” strategy implies the death of the main stressed population (e.g., in the biofilm), where a few viable surviving bacteria can resume growth and restore the original population, once the environmental conditions are suitable. Persister cells and antibiotic heteroresistance are the main causes of recurrent and difficult-to-eradicate infections. Huge progress was made in the last decade toward understanding the mechanisms underlying these processes. However, there are still several issues that remain to be elucidated: (1) the controversy about the definition and metabolic state of persister cells still exists, and multiple definitions of heteroresistance used in the literature may often lead to confusing and inconsistent conclusions [228]; (2) furthermore, different types of heteroresistant or persister cells, including viable but non-culturable bacteria and L-forms, may coexist in the same population; (3) persisters and heteroresistance are difficult to detect or diagnose with standard procedures; (4) it should also be kept in mind that most data regarding antibiotic persistence and heteroresistance originate from experiments performed in laboratory settings and animal models; therefore, they may not reflect the fate of pathogen cells in the human host; (5) inappropriate use of drugs may cause rapid development of persistence and resistance. These problems are associated with most pathogenic infections, but they are particularly important in the case of *A. baumannii,* due to its nosocomial origin and dramatically increasing prevalence of MDR isolates. Antibiotic persistence, population heterogeneity, and biofilm-related resistance should be considered as significant risk factors in the course of choosing an appropriate therapy. In the past few years, several strategies that eliminate *A. baumannii* biofilms and kill persister cells were reported. These approaches include the combination of antibiotics, natural or synthetic AMPs, stringent response inhibitors, QS antagonists, and biofilm disruptors [149,236,237,238,239,240,241,242,243,244,245]. Although these results are promising, further studies are needed to implement novel therapeutic strategies or design drug candidates that will effectively combat *A. baumannii* infections.

## Abbreviations

AHL	*N*-Acyl homoserine lactone
AMEs	Aminoglycoside-modifying enzymes
AMP	Antimicrobial peptide
APRs	Aggregation-prone sequences
BZK	Benzalkonium chloride
CPS	Capsular polysaccharides
DCs	Dendritic cells
EPS	Extracellular polymeric substances
ICU	Intensive care units
IHF	Integration host factor
IM	Inner membrane
LOS	Lipooligosaccharides
LPS	Lipopolysaccharides
MDR	Multidrug-resistant
NETs	Neutrophil extracellular traps
OM	Outer membrane
OMP	Outer membrane protein
OMVs	Outer membrane vesicles
PAMPs	Pathogen-associated molecular patterns
PDR	Pan-drug resistant
PNAG	Poly-β-(1–6)-*N*-acetylglucosamine
PRRs	Pattern recognition receptors
QACs	Quaternary amine compounds
QS	Quorum-sensing
QQ	Quorum-quenching
ROS	Reactive oxygen species
RRF	Ribosomal recycling factor
T2SS	Type II secretion system
TLR4	Toll-like receptor 4
TNF	Tumor necrosis factor
XDR	Extensively drug-resistant
